# Differences in cytotoxicity and apparent extracellular glucose utilisation among sequentially prepared *Cinnamomum cassia* stem-bark extracts in basal and palmitate-associated C2C12 myotubes

**DOI:** 10.3389/fphar.2026.1971518

**Published:** 2026-10-28

**Authors:** Hlayiseka Shikwambana, Ntwanano Sipho Mapfumari, Sebolaishi Makhubela, Mopeledi Madihlaba, Phineas Mokgotho, Leshweni Jeremia Shai, Olebogeng Majane, Mahlogonolo Phasha, Daniel Tswaledi, Sechene Gololo, Ananias Kgopa

**Affiliations:** 1 Department of Biochemistry and Biotechnology, School of Science and Technology, Sefako Makgatho Health Sciences University, Pretoria, South Africa; 2 Department of Physiology, School of Medicine, Sefako Makgatho Health Sciences University, Pretoria, South Africa; 3 Department of Biomedical Sciences, Tshwane University of Technology, Pretoria, South Africa

**Keywords:** apparent extracellular glucose utilisation, C2C12 skeletal muscle cells, *Cinnamomum cassia*, cytotoxicity, palmitate-associated metabolic stress, phytochemical screening, sequential extraction

## Abstract

**Background:**

*Cinnamomum cassia* bark has a documented history of use in traditional medicine and food-medicine practices associated with diabetes and metabolic disorders. This study compared preliminary phytochemical fingerprints, MTT metabolic-viability responses and apparent extracellular glucose utilisation among sequentially prepared stem-bark extracts.

**Methods:**

Authenticated, vouchered stem bark was extracted sequentially with n-hexane, ethyl acetate, ethanol and water. Extracts were examined by thin-layer chromatography and qualitative phytochemical screening. MTT reduction was assessed after 72 h. Residual extracellular glucose was measured after 180 min, and apparent extracellular glucose utilisation was calculated as 15 mmol/L minus the residual concentration. This endpoint does not directly measure membrane glucose transport or intracellular utilisation. Three independent experiments were conducted in technical triplicate, with technical wells averaged within experiments. The retained non-insulin basal well-level values were reconstructed from reported summary statistics and were therefore treated descriptively; the palmitate-associated dataset was analysed using independent-experiment means.

**Results:**

The sequential fractions showed different preliminary fingerprints and concentration-related MTT profiles. Within each palmitate-associated extract series, one or more concentrations showed greater apparent extracellular glucose utilisation than the concurrent palmitate-only control; no formal comparison among extracts was performed.

**Conclusion:**

Sequential fractions showed different MTT and apparent extracellular glucose-utilisation profiles. The endpoint is indirect, was not normalised to concurrent viability or cell number, and may be affected by vehicle or assay interference. Quantitative cinnamaldehyde and coumarin characterisation was not performed. The findings are hypothesis-generating and do not establish glucose uptake, GLUT4 activation, insulin sensitisation, extract superiority, therapeutic efficacy or *in vivo* safety.

## 1 Introduction

Type 2 diabetes mellitus (T2DM) is a complex and progressive metabolic disorder characterised by chronic hyperglycaemia resulting from impaired insulin secretion, insulin resistance, or both, leading to disrupted glucose homeostasis and severe metabolic complications (Galicia-Garcia et al., 2020). The global prevalence of T2DM continues to rise because of increasing obesity, sedentary lifestyles, population ageing, and unhealthy dietary habits, making it one of the leading causes of morbidity and mortality worldwide (Singh et al., 2024; Garg, 2025). Persistent hyperglycaemia contributes to microvascular and macrovascular complications, including diabetic nephropathy, neuropathy, retinopathy and cardiovascular disease (Galicia-Garcia et al., 2020; Susilawati et al., 2023). Skeletal muscle is a major site of insulin-stimulated glucose disposal (Sylow et al., 2021; Richter et al., 2025). Under physiological conditions, insulin-receptor activation recruits insulin receptor substrate, phosphoinositide 3-kinase and protein kinase B signalling, promoting glucose transporter type 4 translocation to the plasma membrane and glucose transport (Sylow et al., 2021; Knudsen et al., 2020; Herman et al., 2022; Deenadayalan et al., 2021; Jaiswal et al., 2026; Richter et al., 2025). Impairment of this pathway contributes to skeletal-muscle insulin resistance (Sylow et al., 2021; Knudsen et al., 2020; Herman et al., 2022; Deenadayalan et al., 2021; Richter et al., 2025). These established mechanisms provide the physiological context for investigating glucose handling, but the extracellular-glucose-disappearance assay used here does not measure this transport pathway directly.

Although conventional antidiabetic medicines, including metformin, sulfonylureas, thiazolidinediones and insulin, improve glycaemic control, long-term treatment can be limited by adverse effects, reduced effectiveness, poor adherence, and progressive treatment failure (Susilawati et al., 2023). These limitations have encouraged investigation of medicinal plants containing structurally diverse compounds that may modulate glucose metabolism, oxidative stress, inflammation, and insulin signalling (Oluwole et al., 2022; Khaafi et al., 2024; Lee et al., 2025; Kang et al., 2019; Pannucci et al., 2023; Krawczyk et al., 2023). *C. cassia* is widely investigated for its traditional use in diabetes and other metabolic disorders (Nair, 2021; Khaafi et al., 2024; Pandey et al., 2020; Haldar et al., 2022). Its stem bark contains cinnamaldehyde, flavonoids, phenolic compounds, terpenoids, tannins, coumarins, glycosides and cinnamic acid derivatives with reported antioxidant, anti-inflammatory and glucose-lowering properties (Nair, 2021; Khaafi et al., 2024; Pandey et al., 2020; Haldar et al., 2022; Guo et al., 2024; Karimirad et al., 2025; Zhang et al., 2026). Cinnamon extracts and constituents have also been reported to influence skeletal-muscle glucose handling and related metabolic signalling pathways (Khaafi et al., 2024; Mohsin et al., 2023; Guo et al., 2024; Karimirad et al., 2025; Zhang et al., 2026; Shen et al., 2014; Absalan et al., 2012; Nikzamir et al., 2014; Lakshmi et al., 2009; Gannon et al., 2015).

*Cinnamomum cassia* bark has a long history of use in Chinese and other East Asian traditional medicine systems and in food-medicine practices, including applications associated with diabetes and metabolic disorders (Zhang et al., 2026; Zhang et al., 2019). The present pharmacological question was therefore grounded in documented traditional use and in prior work on the same authenticated stem-bark material (Kgopa et al., 2022). Although the commercial material was sourced from an Indigenous Knowledge Systems practitioner in Pretoria, no formal ethnobotanical interview was undertaken; accordingly, this study does not attribute a distinct South African traditional indication to the supplied material. The sequential n-hexane, ethyl acetate, ethanol and aqueous procedure was an analytical fractionation strategy and was not intended to reproduce or validate a traditional preparation.

Solvents of different polarity recover chemically different subsets of complex plant material (Singarayar et al., 2025; El-Saadony et al., 2025; Sun et al., 2025; Ramesh et al., 2024; Kozhantayeva et al., 2024; Torres-Vega et al., 2020; Al-Nemi et al., 2022; Lee et al., 2026; Bouaita et al., 2026; Sharifi et al., 2025). Non-polar solvents such as n-hexane generally favour lipophilic constituents, whereas ethyl acetate, ethanol, and water favour different polar constituents; however, polarity alone does not establish extract composition or biological causation (Singarayar et al., 2025; El-Saadony et al., 2025; Sun et al., 2025; Ramesh et al., 2024; Kozhantayeva et al., 2024; Torres-Vega et al., 2020; Al-Nemi et al., 2022; Lee et al., 2026; Bouaita et al., 2026; Sharifi et al., 2025). Direct comparisons of sequentially prepared *C. cassia* stem-bark fractions under basal and palmitate-associated metabolic stress remain limited. This study therefore compared the preliminary phytochemical fingerprints, cytotoxicity and apparent extracellular glucose utilisation associated with sequential fractions prepared using n-hexane, ethyl acetate, ethanol, and water. The work was designed as a screening study to prioritise fractions for quantitative chemical characterisation and direct transport and signalling experiments.

The novelty of the present work is not a first demonstration that cinnamon-related preparations can influence skeletal-muscle glucose biology. Previous studies reported that cinnamon extracts increased glucose uptake through LKB1-AMPK signalling in C2C12 myotubes, hydro-alcoholic cinnamon extract increased membrane-associated GLUT4, cinnamaldehyde increased GLUT4 expression in C2C12 cells, and cinnamic acid influenced GLUT4-related glucose transport in L6 myotubes (Shen et al., 2014; Absalan et al., 2012; Nikzamir et al., 2014; Lakshmi et al., 2009). Trans-cinnamaldehyde has also been reported to increase GLUT4-related metabolic-gene expression while reducing myotube oxidative metabolism, illustrating that marker expression and functional metabolism need not change in parallel (Gannon et al., 2015). The narrower contribution of this study is the comparison of sequential *C. cassia* fractions using the same C2C12 screening workflow under basal and palmitate-associated conditions. Clinical evidence also remains heterogeneous: an earlier meta-analysis found no significant improvement in glycated haemoglobin or fasting glucose, whereas a recent meta-analysis reported improvements in several glycaemic indices (Baker et al., 2008; Moridpour et al., 2024). These differences preclude clinical extrapolation from the present *in vitro* observations.

## 2 Materials and methods

### 2.1 Study design

This study compared sequentially prepared *Cinnamomum cassia* stem-bark extracts obtained using n-hexane, ethyl acetate, ethanol, and distilled water. The workflow comprised sequential extraction; TLC fingerprinting and qualitative phytochemical screening; MTT cytotoxicity testing; measurement of apparent extracellular glucose utilisation under basal conditions and during continued palmitate-associated metabolic stress; and within-extract statistical analysis using independent-experiment means.

### 2.2 Plant material collection and identification

Commercial stem bark material sold as *C*. *cassia* was obtained from Mr B. Maswanganyi, a local vendor and Indigenous Knowledge Systems practitioner in Pretoria, Gauteng, South Africa. The botanical identity of the material was authenticated by the South African National Biodiversity Institute. A voucher specimen was deposited in the School of Pharmacy Herbarium, Sefako Makgatho Health Sciences University, Pretoria, South Africa, under voucher number CINS04. The authenticated material was air-dried, ground to a fine powder using a Polymix DX-MFC 90D homogeniser (Kinematica, Italy; mesh size 200), and stored in airtight containers until extraction.

### 2.3 Preparation of plant extracts

Powdered stem bark was extracted sequentially using n-hexane, ethyl acetate, ethanol, and distilled water. For each organic solvent, the plant material was macerated with agitation and filtered through Whatman No. 1 filter paper. The extraction step was repeated, and the combined organic filtrates were concentrated under reduced pressure using a Buchi rotary evaporator. For the aqueous fraction, the residual plant material was boiled in distilled water for 2 h, filtered and freeze-dried. The dried extracts were stored at 4 °C until use. Stock solutions were prepared at 20 mg/mL in 100% dimethyl sulfoxide (DMSO) and diluted freshly in the relevant assay medium. The retained records available for this revision did not permit independent confirmation of the starting plant mass, solvent-to-solid ratio, duration of each organic-solvent extraction, detailed agitation schedule or the underlying yield-calculation worksheet. These omissions limit exact replication and are acknowledged rather than reconstructed.

Natural-product reporting followed the ConPhyMP principles to the extent supported by the retained records. The accepted species name, plant part, source, authentication authority, voucher number, processing and sequential solvent order are reported. Exact starting mass, solvent-to-solid ratio, organic-extraction duration, and detailed agitation schedule were not recoverable from the records supplied for this revision. Reported extraction yields are retained from the original dataset, but the underlying calculation worksheet was not available for verification. Solvent polarity, extraction order and depletion of the residual plant matrix are interrelated in this sequential design and cannot be separated causally. TLC and colour-reaction screening provide preliminary comparative fingerprints only; they do not identify individual compounds or provide quantitative standardisation.

### 2.4 Thin-layer chromatography (TLC) fingerprinting

Thin-layer chromatography (TLC) was performed to compare the preliminary phytochemical fingerprints of the sequentially prepared n-hexane, ethyl acetate and ethanol *C. cassia* stem-bark extracts. Aluminium-backed silica gel 60 F254 TLC plates (Merck, Darmstadt, Germany) were used as the stationary phase. Each extract was prepared at 10 mg/mL, and approximately 10 µL was applied as narrow bands using glass capillary tubes and air-dried at room temperature. Chromatographic separation was performed using three mobile phases: benzene:ethanol:ammonium hydroxide (BEA; 90:10:1, v/v/v), chloroform:ethyl acetate:formic acid (CEF; 5:4:1, v/v/v), and ethyl acetate:methanol:water (EMW; 40:5.4:5, v/v/v). Plates were developed separately in pre-saturated chambers until the solvent front had migrated approximately 8 cm from the point of application.

Following development, the chromatograms were air-dried and initially examined under ultraviolet (UV) light at 254 nm and 365 nm to detect UV-active and fluorescent compounds. The plates were subsequently derivatised separately with anisaldehyde and vanillin-sulphuric acid reagents, followed by heating at 105 °C for 5–10 min to enhance the visualisation of phytochemical constituents. The number, colour, intensity, and migration patterns of the resolved bands were recorded and compared among the solvent extracts. Retention factor (Rf) values were calculated using the following equation:

$$R_{f}=\frac{d_{c}}{d_{f}}$$

Where *R*_*f*_ is the retention factor; *d*_*c*_ is the distance travelled by the centre of the compound band from the application origin; and *d*_*f*_ is the distance travelled by the solvent front from the same origin. All distances were measured in the same units.

The chromatographic fingerprints generated using the three solvent systems were compared to describe differences in the number, migration and visualisation of bands among the sequentially prepared *C. cassia* stem-bark extracts.

### 2.5 Qualitative phytochemical screening

Qualitative phytochemical screening was performed using standard colour-reaction and precipitation procedures to record the presence or absence of major secondary-metabolite classes (Shaikh and Patil, 2020; Maheshwaran et al., 2024; Nagori et al., 2025; Ezeonu and Ejikeme, 2016). The extracts were screened for alkaloids, flavonoids, phenolic compounds, tannins, terpenoids, steroids, saponins, glycosides, coumarins and anthraquinones. These class-level tests were interpreted as preliminary screening and not as compound identification or quantitative analysis.

### 2.6 Cell culture

Murine C2C12 skeletal muscle myoblasts (ATCC® CRL-1772™) were cultured in Dulbecco’s Modified Eagle Medium (DMEM; Gibco, Thermo Fisher Scientific, United States) supplemented with 10% heat-inactivated fetal bovine serum and 1% penicillin–streptomycin, providing final concentrations of 100 U/mL penicillin and 100 μg/mL streptomycin. Cells were maintained at 37 °C in a humidified atmosphere containing 5% CO_2_ and were subcultured at approximately 80% confluence. Cells between passages 5 and 20 were used for all experiments. For the experimental assays, C2C12 myoblasts were seeded in tissue-culture-treated flat-bottom 96-well plates at a density of 1 × 10^5^ cells/mL. A 100-µL cell suspension was added to each well, corresponding to 1 × 10^4^ cells/well. The cells were incubated for 24 h to permit attachment. Differentiation was induced by replacing the growth medium with DMEM supplemented with 2% horse serum. Cells were differentiated for 5 days until multinucleated myotubes were formed, with the differentiation medium replaced every 48 h. Cultures were routinely examined microscopically for normal morphology, growth characteristics and visible microbial contamination. Specific testing for *mycoplasma* contamination was not performed.

### 2.7 MTT metabolic-viability assay

The effects of the sequentially prepared *Cinnamomum cassia* stem-bark extracts on C2C12 cell viability were determined using the 3-(4,5-dimethylthiazol-2-yl)-2,5-diphenyltetrazolium bromide (MTT) assay. C2C12 myoblasts were seeded in tissue-culture-treated flat-bottom 96-well plates at a density of 1 × 10^5^ cells/mL, with 100 µL of cell suspension added to each well, corresponding to 1 × 10^4^ cells/well. The cells were allowed to attach for 24 h and were subsequently differentiated *in situ* for 5 days. The original method recorded exposure to n-hexane, ethyl acetate, ethanol or aqueous fractions at 1.6–200 μg/mL for 72 h. However, the retained source figure and numerical records available for this revision covered 1.6, 3.125, 6.25, 12.5, 25, 50 and 100 μg/mL; the 200-µg/mL observations could not be verified and are not presented. Extract stocks were prepared at 20 mg/mL in 100% DMSO; direct dilution to the stated range produced nominal DMSO fractions of 0.008%–1.0% (v/v). Untreated cells served as the negative control. A concurrent 1% (v/v) DMSO control matched the maximum extract-associated DMSO fraction, whereas H_2_O_2_ at 1% (v/v) served as the cytotoxic positive control, consistent with the previously published method for these *C. cassia* stem-bark extracts (Kgopa et al., 2022). All controls were exposed for the same 72-h treatment period as the extracts.

Following treatment, 20 µL of MTT solution at 5 mg/mL was added to each well, and the plates were incubated for 4 h at 37 °C. The medium was then carefully aspirated, and the resulting formazan crystals were dissolved in 100 µL of DMSO. Absorbance was measured at 570 nm using a SpectraMax iD3 microplate reader (Molecular Devices, San Jose, CA, United States). Cell-free interference controls containing culture medium, the corresponding concentration of each extract and MTT reagent, but no cells, were included at every tested concentration. The absorbance of each extract only blank was subtracted from that of the corresponding cell-containing treatment wells to correct for background absorbance associated with extract colour, turbidity, or direct interaction with the MTT reagent. Cell viability was calculated using the following equation:

$$\text{Cell viability }\left(\%\right)=\frac{A_{\text{treatment}}-A_{\text{blank}}}{A_{\text{control}}-A_{\text{blank}}}\times 100$$

Where *A*_*treatment*_ is the absorbance at 570 nm of cells exposed to the extract; *A*_*blank*_ is the absorbance of the corresponding cell-free extract blank; and *A*_*control*_ is the absorbance of the untreated or matched vehicle-control cells, as applicable.

Three independent experiments were conducted, with every condition tested in technical triplicate. Technical wells were averaged within each independent experiment. Complete experiment-level nonlinear-regression outputs, Hill slopes, goodness-of-fit statistics and curve-level confidence intervals were not retained. Because the earlier IC_50_ estimates could not be audited against those source fits, the estimates have been withdrawn from this revision. The MTT results are therefore presented directly as concentration-related metabolic-viability profiles rather than as fitted potency parameters.

### 2.8 Induction of palmitate-associated metabolic stress

Palmitate-associated metabolic stress was induced using sodium palmitate complexed with fatty-acid-free bovine serum albumin (BSA). A 100 mM sodium palmitate stock in 70% (v/v) ethanol was added dropwise at 1:20 (v/v) to warmed 10% (w/v) fatty-acid-free BSA and mixed at 55 °C for 10–15 min to permit non-covalent fatty-acid binding. The preparation was adjusted to pH 7.0–7.4, aliquoted and stored at −20 °C until use.

The complex was diluted in serum-free DMEM to 0.5 mM palmitate, yielding approximately 1.0% (w/v) final BSA and 0.35% (v/v) final ethanol, and was sterilised through a 0.22-µm membrane filter before use. BSA-only and BSA plus 0.35% (v/v) ethanol vehicle controls were prepared and run in parallel. Differentiated C2C12 myotubes were exposed to 0.5 mM palmitate–BSA for 16 h. Palmitate-containing medium was retained during the subsequent treatment so that responses were assessed under continued palmitate-associated metabolic stress rather than after palmitate withdrawal.

### 2.9 Apparent extracellular-glucose-utilisation assay

Apparent extracellular glucose utilisation was measured using a glucose oxidase-based colorimetric assay of residual extracellular glucose. The endpoint was calculated as the difference between the starting glucose concentration and the residual concentration in collected medium. It estimates glucose disappearance from the medium but does not by itself identify the membrane-transport pathway, intracellular fate of glucose or GLUT4 activity. C2C12 myoblasts were seeded in tissue-culture-treated flat-bottom 96-well plates at 1 × 10^5^ cells/mL, with 100 µL per well (1 × 10^4^ cells/well), allowed to attach and differentiated for 5 days in DMEM supplemented with 2% horse serum.

Differentiated myotubes were washed twice with phosphate-buffered saline and serum-starved for 2 h. The cells were then washed twice with glucose-free Krebs–Ringer bicarbonate HEPES buffer and equilibrated for 30 min at 37 °C in the same buffer supplemented with 0.1% (w/v) BSA. The assay was subsequently conducted in buffer containing 15 mM D-glucose. The sequentially prepared n-hexane, ethyl acetate, ethanol and aqueous *C. cassia* stem-bark fractions were tested at 0.16, 0.31, 0.63, 1.25, 2.50 and 5.00 μg/mL. Stocks were prepared at 20 mg/mL in 100% DMSO, corresponding to nominal final DMSO fractions of approximately 0.0008%–0.025% (v/v). The original workflow specified matched vehicle controls; however, concentration-specific DMSO-control results for the 180-min functional assay were not available in the records supplied for this revision. Consequently, an effect of DMSO on extracellular glucose utilisation cannot be excluded.

The 0.16–5.00 μg/mL range was selected as a low-concentration functional screening range within the concentrations examined in the 72-h MTT experiment; it was not derived from pharmacokinetic or clinical-exposure data. The highest n-hexane functional concentration approached the range in which the retained 72-h MTT profile fell below 50%, whereas the functional exposure lasted 180 min. Viability, cell number and protein content were not measured concurrently during the functional assay. The tested concentrations therefore do not define a therapeutic or definitive *in vitro* safety window, and altered cell integrity, viable cell number or metabolic stress cannot be excluded as contributors to extracellular glucose disappearance. For the palmitate-associated condition, differentiated C2C12 myotubes were exposed to 0.5 mM palmitate-BSA for 16 h. The palmitate-containing medium was retained during a further 180-min treatment with extracts or metformin (2 mM). A concurrent insulin-plus-palmitate condition was included, but the insulin concentration and exposure duration were not documented in the retained palmitate source record. Concurrent groups comprised the normal control, palmitate-only control, insulin plus palmitate, metformin plus palmitate and palmitate plus extract. These controls establish the comparison structure but do not validate insulin resistance or a specific insulin-signalling mechanism.

Following treatment, extracellular medium was collected, and residual glucose was quantified using a glucose oxidase-based colorimetric kit supplied by KAT Medical Laboratory, South Africa. For each determination, 10 µL of sample or glucose standard was mixed with 1.0 mL of working reagent, incubated for 5 min at 37 °C and measured at 492 nm against a reagent blank. A 5.56 mmol/L glucose standard was used to calculate residual extracellular glucose. The retained method documented a reagent blank but did not document concentration-specific cell-free extract blanks, glucose spike-recovery experiments or another orthogonal interference assessment. Extract colour, turbidity or chemical interaction with the glucose oxidase/peroxidase reaction therefore cannot be excluded.

The residual glucose concentration was calculated as follows:

$$C_{\text{residual}}\;\left(\text{mmol}/L\right)=\frac{A_{\text{sample}}}{A_{\text{standard}}}\times 5.56\text{ mmol}/L$$

Where *C*_*residual*_ is the residual extracellular glucose concentration; *A*_*sample*_ is the absorbance at 492 nm of the test sample; *A*_*standard*_ is the absorbance at 492 nm of the glucose standard; and 5.56 mmol/L is the concentration of that standard.

Apparent extracellular glucose utilisation was calculated as:

$$U=C_{0}-C_{\text{residual}}=15\text{ mmol}/L\;‐\;C_{\text{residual}}$$

Where *U* is apparent extracellular glucose utilisation (mmol/L); *C*_*0*_ is the starting extracellular glucose concentration (15 mmol/L); and *C*_*residual*_ is the residual extracellular glucose concentration after treatment (mmol/L).

The percentage change relative to the untreated control under basal conditions was calculated as:

$$\text{Percentage change }\left(\%\right)=\frac{U_{\text{treated}}-U_{\text{untreated}}}{U_{\text{untreated}}}\times 100$$

Where *U*_*treated*_ is apparent extracellular glucose utilisation in extract-treated cells, and *U*_*untreated*_ is apparent extracellular glucose utilisation in untreated control cells. Both values are expressed in mmol/L.

For the palmitate-associated condition, the percentage change relative to the palmitate-only mean was calculated as:

$$\text{Percentage change relative to palmitate }\left(\%\right)=\frac{U_{\text{treatment}}-U_{\text{palmitate}}}{U_{\text{palmitate}}}\times 100$$

Where *U*_*treatment*_ is the mean apparent extracellular glucose utilisation in the treatment group, and *U*_*palmitate*_ is the mean apparent extracellular glucose utilisation in the palmitate-only group. Both values are expressed in mmol/L.

Three independent experiments were conducted, with each condition tested in technical triplicate. Technical wells were averaged within each independent experiment; the three independent-experiment means constituted the biological replicates for inference.

### 2.10 Statistical analysis

Three independent experiments were conducted, each comprising three technical wells. Technical wells were averaged within each experiment. For the palmitate-associated dataset, the three independent-experiment means were the units of analysis (biological n = 3). Separate one-way ANOVAs were retained within each extract block and followed by Tukey’s honestly significant difference test. To control the four primary extract-block ANOVA families, a Bonferroni-adjusted family threshold of α = 0.0125 (0.05/4) was applied; all four reported omnibus p values remained below this threshold. Tukey adjustment controlled comparisons within each block. This analysis does not test an extract-by-concentration interaction, and no cross-extract ranking is inferred. A mixed-effects or factorial model was not fitted because biological n = 3 would not support stable interaction and random-effect estimation. For the basal dataset, non-insulin well-level values available for reanalysis had been mathematically reconstructed from reported summary statistics; basal extract comparisons were therefore treated as descriptive and were not subjected to confirmatory inference.

## 3 Results

### 3.1 Percentage extraction yield of *C. cassia* stem-bark extracts

The reported extraction yields of the sequentially prepared *C. cassia* stem-bark fractions are presented in Figure 1 ethanol, 2.88%; ethyl acetate, 2.08%; n-hexane, 1.62%; and water, 1.52%. These values are retained from the original dataset, but the underlying worksheet documenting the starting dry mass and calculation was not available for independent verification. They should not be interpreted as independent solvent extraction efficiencies because later solvents acted on material depleted by earlier extraction steps.

**FIGURE 1:**
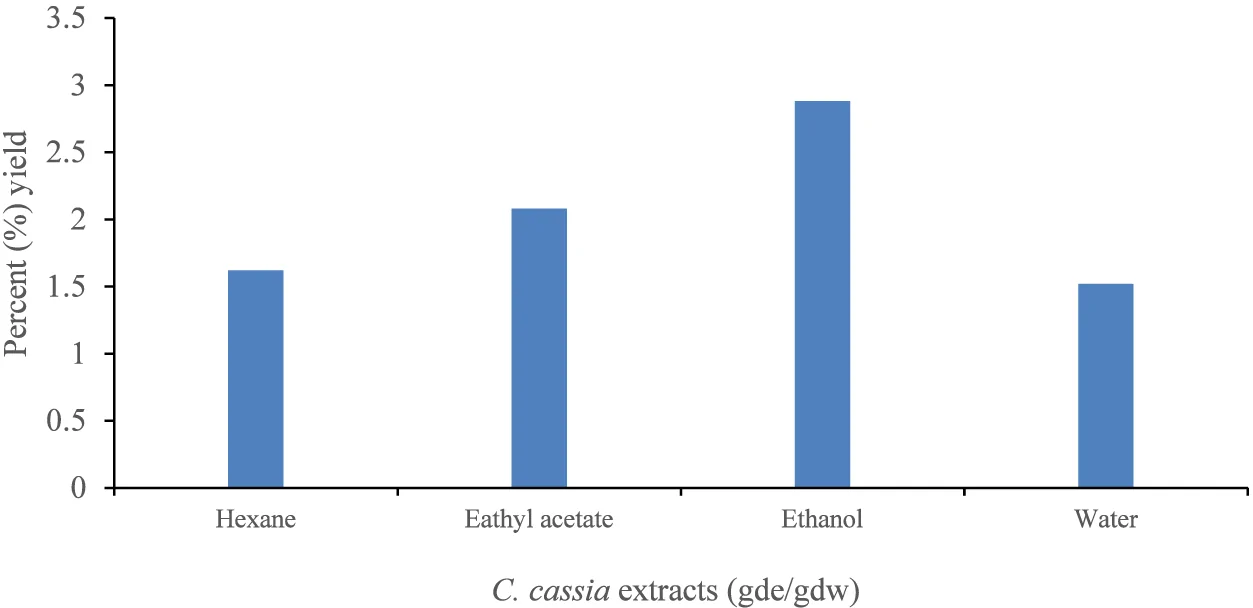
Reported percentage extraction yields of sequentially prepared *C. cassia* stem-bark fractions. The values are retained from the original dataset. The underlying worksheet documenting the starting dry mass and yield calculation was not available for independent verification during this revision.

### 3.2 Thin-layer chromatography (TLC) fingerprinting of *C. cassia* stem-bark extracts

Distinct chromatographic profiles were observed among the sequentially prepared n-hexane, ethyl acetate and ethanol extracts (Figure 2). Representative retention-factor values and descriptive band observations are summarised in Table 1. The three mobile phases produced different visual band patterns. Anisaldehyde derivatisation revealed 49 bands across the reported chromatograms (25 in BEA, 15 in CEF and 9 in EMW), whereas vanillin-sulphuric acid revealed 46 bands (25 in CEF, 15 in BEA and 6 in EMW). These counts represent chromatographic bands rather than identified chemical compounds. Several bands had matching Rf values across extracts and may represent shared constituents, although identity cannot be inferred from Rf alone. Bands at Rf 0.54 in EMW were observed in the n-hexane, ethyl acetate and ethanol extracts after both derivatisation procedures; bands at Rf 0.98 in CEF and 0.96 in BEA were also shared across the three extracts. Overall, the band number, migration and visualisation patterns provided preliminary descriptive fingerprints of the sequential extracts; they did not establish extraction efficiency, phytochemical abundance or chemical diversity.

**TABLE 1:** Representative retention factor (Rf) values of phytochemical constituents detected in sequentially prepared *Cinnamomum cassia* stem-bark extracts separated using EMW, CEF and BEA solvent systems.

Solvent system	Extract	Representative Rf values observed*	Bands observed	Descriptive observation
BEA (benzene:ethanol:ammonium hydroxide, 90:10:1)	n-hexane	0.43, 0.69, 0.96	Fewer coloured bands than ethyl acetate and ethanol extracts	Fewer visible bands than ethyl acetate and ethanol
​	Ethyl acetate	0.16, 0.25, 0.30, 0.43, 0.69, 0.96	Greater number of coloured bands than n-hexane	More visible bands than n-hexane
​	Ethanol	0.13, 0.25, 0.30, 0.43, 0.69, 0.96	Highest number of coloured bands observed	Most visible bands within the BEA plate
CEF (chloroform:ethyl acetate:formic acid, 5:4:1)	n-hexane	0.53, 0.58, 0.65, 0.68, 0.75, 0.83, 0.98	Several coloured bands detected	Several visible bands
​	Ethyl acetate	0.06, 0.40, 0.53, 0.58, 0.65, 0.68, 0.75, 0.83, 0.98	Numerous coloured bands	Numerous visible bands
​	Ethanol	0.06, 0.40, 0.53, 0.58, 0.65, 0.68, 0.75, 0.83, 0.98	Greatest number of coloured bands	Most visible bands within the CEF plate
EMW (ethyl acetate:methanol:water, 40:5.4:5)	n-hexane	0.54, 0.98	Shared bands detected at Rf 0.54	Two representative Rf positions reported
​	Ethyl acetate	0.54, 0.98	Shared bands with ethanol and n-hexane at Rf 0.54	Shared representative band at Rf 0.54
​	Ethanol	0.54, 0.98	Highest number of coloured bands in EMW	Shared representative bands at Rf 0.54 and 0.98

Abbreviations: BEA, benzene: ethanol: ammonium hydroxide; CEF, chloroform: ethyl acetate: formic acid; EMW, ethyl acetate: methanol: water; Rf, retention factor; UV, ultraviolet. *Only representative Rf values are presented. Chromatograms were visualised under UV light (254 and 365 nm) before and after derivatisation with anisaldehyde and vanillin-sulphuric acid. The ethanol extract showed the greatest number of coloured bands, followed by ethyl acetate and n-hexane. Across the reported chromatograms, 49 bands were visualised after anisaldehyde derivatisation (25 in BEA, 15 in CEF and 9 in EMW), and 46 after vanillin-sulphuric acid derivatisation (25 in CEF, 15 in BEA and 6 in EMW). Shared Rf values suggest, but do not establish, common constituents.

**FIGURE 2:**
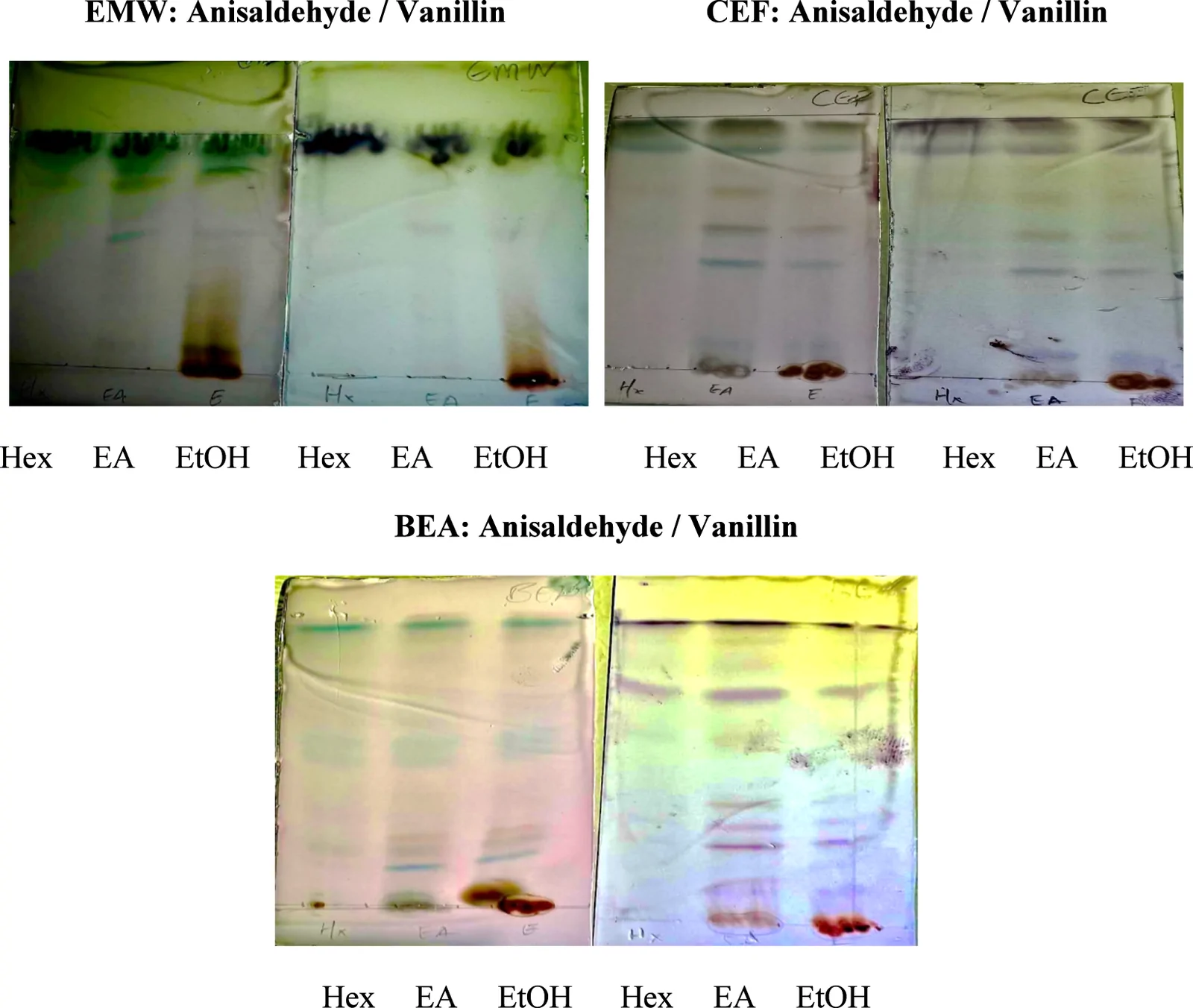
Thin-layer chromatography (TLC) fingerprints of sequentially prepared *C. cassia* stem-bark extracts developed in ethyl acetate:methanol:water (EMW), chloroform:ethyl acetate:formic acid (CEF) and benzene:ethanol:ammonium hydroxide (BEA) solvent systems. Chromatograms were visualised following derivatisation with anisaldehyde and vanillin-sulphuric acid reagents, demonstrating differences in chromatographic band patterns among the n-hexane, ethyl acetate and ethanol fractions.

### 3.3 Qualitative phytochemical composition of *C. cassia* stem-bark extracts

The qualitative phytochemical screening results are presented in Table 2. The extracts produced different presence/absence patterns in class-level colour and precipitation reactions. The ethanol extract had positive reactions for alkaloids, flavonoids, phenolic compounds, tannins, saponins, terpenoids, glycosides and coumarins. The aqueous extract had positive reactions for alkaloids, flavonoids, phenolic compounds, tannins, saponins, glycosides and coumarins, while terpenoid and steroid reactions were negative.

**TABLE 2:** Qualitative phytochemical constituents of sequentially prepared *C. cassia* stem-bark extracts.

Phytochemical class	Test(s) used	n-hexane	Ethyl acetate	Ethanol	Aqueous
Alkaloids	Mayer’s, Dragendorff’s	−	−	+	+
Flavonoids	Shinoda, Alkaline reagent	−	+	+	+
Phenolic compounds	Ferric chloride	−	+	+	+
Tannins	Ferric chloride, Gelatin	−	−	+	+
Saponins	Froth test	−	−	+	+
Terpenoids	Salkowski	+	+	+	−
Steroids	Liebermann-Burchard	+	+	−	−
Glycosides	Keller-Killiani	−	−	+	+
Coumarins	NaOH/UV fluorescence	−	+	+	+

(+), present; (−), absent.

The ethyl acetate extract had positive reactions for flavonoids, phenolic compounds, terpenoids, steroids and coumarins. The n-hexane extract had positive reactions only in the terpenoid and steroid class tests. These preliminary reactions do not identify individual compounds, quantify abundance or assign biological activity to any class.

### 3.4 Cytotoxicity effects of *C. cassia* extracts on C2C12 cells

Relative to untreated and corresponding DMSO vehicle controls, all four extracts reduced the C2C12 MTT metabolic-viability signal in a concentration-related manner after 72 h. The concurrently run 1% (v/v) DMSO control retained 99.93% ± 0.14% of the untreated-control signal, whereas the H_2_O_2_ positive control retained 40.00% ± 0.11% (mean ± SEM; n = 3 independent-experiment means). In the retained 1.6–100 μg/mL source figure (Figure 3) the mean MTT signal fell below 50% of untreated at 6.25 μg/mL for n-hexane, 25 μg/mL for aqueous extract, and 50 μg/mL for ethyl acetate and ethanol. These are observed concentration-profile descriptors, not fitted IC_50_ values or formal between-extract potency comparisons.

**FIGURE 3:**
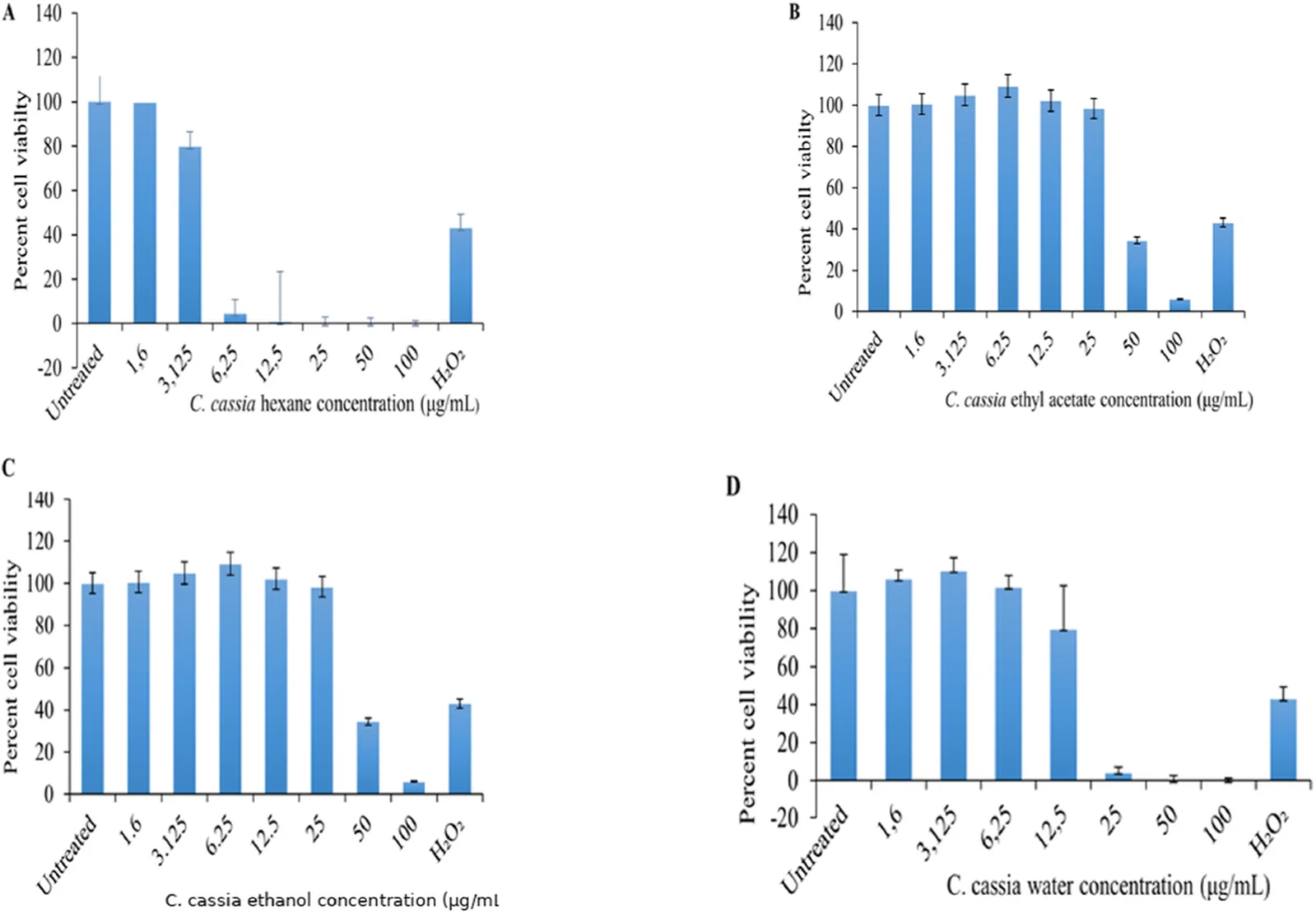
Concentration-dependent effects of sequentially prepared *C. cassia* stem-bark extracts on the C2C12 MTT metabolic-viability signal after 72 h. The retained source figure displays 1.6, 3.125, 6.25, 12.5, 25, 50 and 100 μg/mL and the H_2_O_2_ positive control; the originally reported 200-µg/mL and DMSO-control observations were not available for verification or replotting. Data represent three independent experiments performed in technical triplicate. Panels show **(A)** n-hexane, **(B)** ethyl acetate, **(C)** ethanol and **(D)** aqueous fractions. MTT reduction is interpreted as a metabolic-viability endpoint rather than direct cell number.

### 3.5 Apparent extracellular glucose utilisation under basal conditions

All extracts showed descriptive concentration-related patterns in apparent extracellular glucose utilisation under basal conditions. A descriptive summary of the reported basal responses is presented in Figure 4. Reported maxima were 14.3 ± 0.5 mmol/L for n-hexane at 2.50 μg/mL, 13.4 ± 0.5 mmol/L for ethyl acetate at 1.25 μg/mL, 12.0 ± 0.5 mmol/L for aqueous extract at 2.50 μg/mL and 10.1 ± 0.3 mmol/L for ethanol at 1.25 μg/mL, compared with an untreated mean of 7.4 ± 0.3 mmol/L. Only the insulin wells were retained as observed absorbance values in the supplied basal workbook; the non-insulin well-level values were reconstructed from reported means and standard deviations (SDs). Accordingly, these basal patterns are descriptive and should not be represented as raw observations or used for confirmatory inference.

**FIGURE 4:**
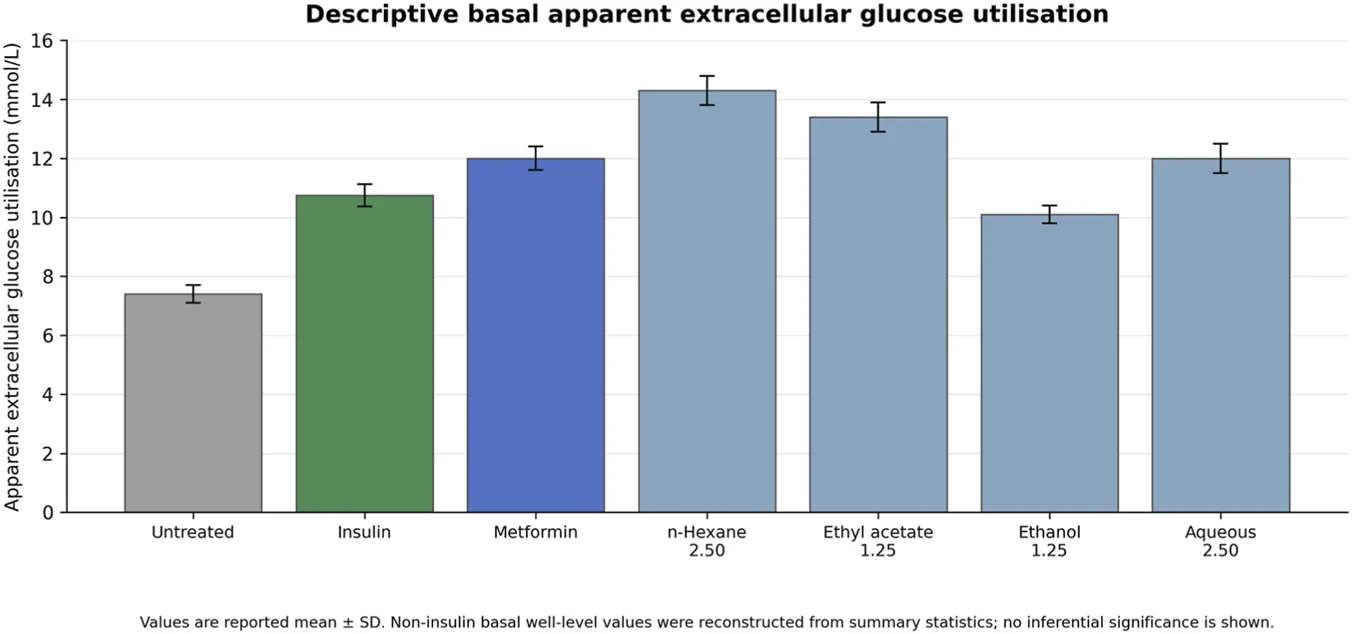
Descriptive summary of basal apparent extracellular glucose utilisation associated with sequentially prepared *C. cassia* stem-bark fractions. Bars show reported mean ± standard deviation for untreated, insulin and metformin controls and the reported maximum response for each extract. Residual extracellular glucose was measured by a glucose oxidase assay, and apparent extracellular glucose utilisation was calculated as starting minus residual extracellular glucose. Only the insulin wells were retained as observed absorbance values in the supplied basal workbook; non-insulin well-level values were mathematically reconstructed from reported means and standard deviations. Reconstructed values are not raw observations, and no confirmatory inferential interpretation is made.

### 3.6 Apparent extracellular glucose utilisation during palmitate-associated metabolic stress

The apparent extracellular glucose utilisation responses during continued palmitate exposure are presented in Figure 5. Exposure to 0.5 mM palmitate-BSA for 16 h was associated with lower apparent extracellular glucose utilisation than the normal control in the retained palmitate-associated dataset. The insulin-plus-palmitate condition produced a recorded response, but its insulin concentration and exposure duration were not documented in the retained source record. It is therefore used only as an incompletely documented assay-response control and not as a mechanistic benchmark. The largest observed mean within each extract series was 10.472 ± 0.289 mmol/L for n-hexane at 2.50 μg/mL, 9.166 ± 0.231 mmol/L for ethyl acetate at 2.50 μg/mL, 8.469 ± 0.425 mmol/L for aqueous extract at 2.50 μg/mL and 6.770 ± 0.155 mmol/L for ethanol at 1.25 μg/mL (mean ± SD; biological n = 3). Approximate unadjusted t-based 95% confidence intervals for these selected means were 9.754–11.190, 8.592–9.740, 7.413–9.525 and 6.385–7.155 mmol/L, respectively. Percentage differences relative to the corresponding palmitate-only mean were 144.63%, 115.10%, 99.35% and 58.35%, respectively. These concentrations were selected *post hoc* because they produced the largest numerical mean in each series and are therefore subject to winner’s-curse bias; the estimates and confidence intervals are exploratory, were not adjusted for that selection and do not identify optimal concentrations. The percentages are descriptive effect sizes relative to the palmitate-only mean and do not imply recovery, return to normal physiology or reversal of metabolic impairment. Separate one-way ANOVAs were significant at the Bonferroni-adjusted family threshold within all four extract blocks, but these analyses do not provide a formal comparison among extracts.

**FIGURE 5:**
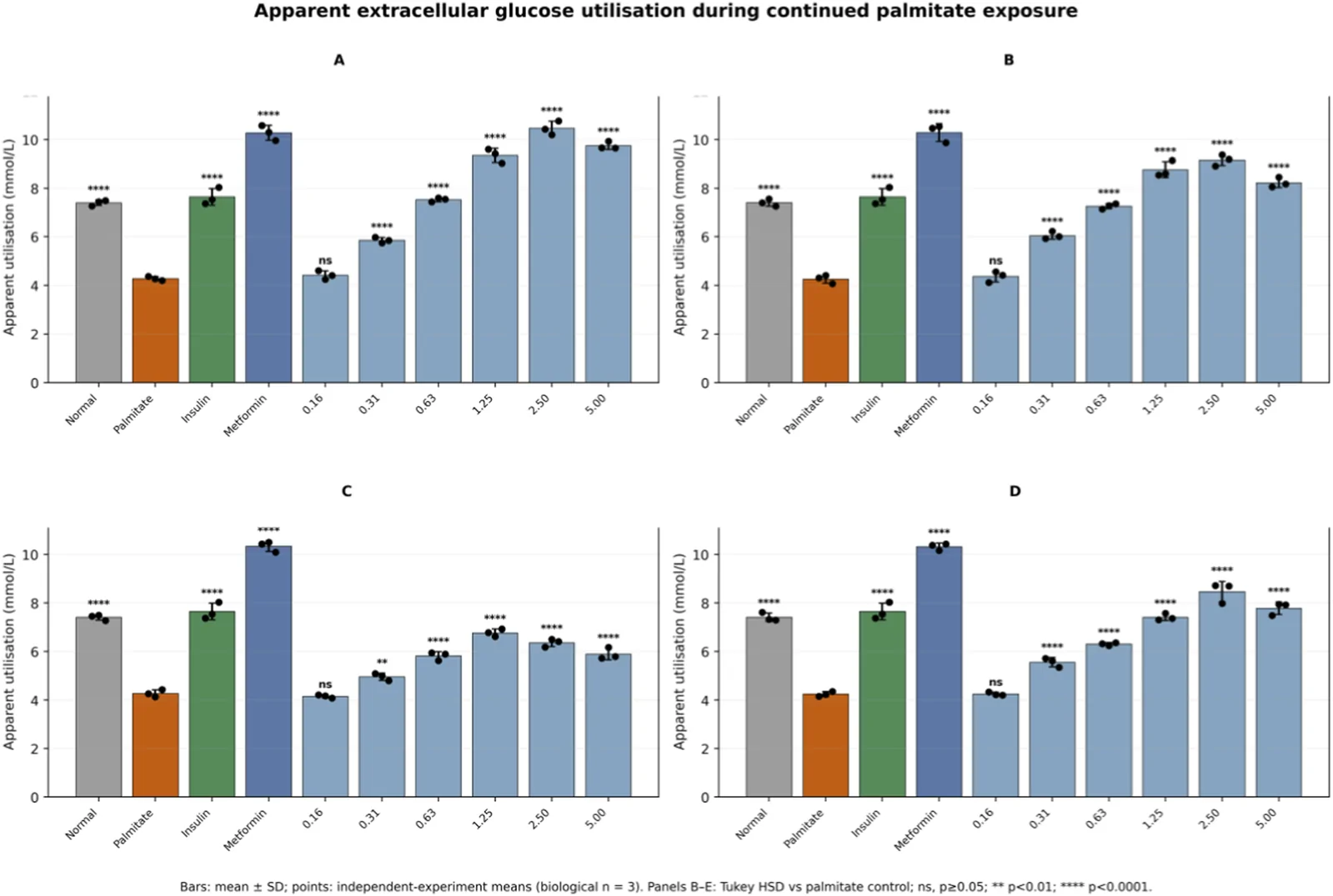
Apparent extracellular glucose utilisation in the retained palmitate-associated dataset. Myotubes were exposed to 0.5 mM palmitate for 16 h and then treated during continued palmitate exposure. Panels show **(A)** n-hexane, **(B)** ethyl acetate, **(C)** ethanol and **(D)** aqueous extracts. Bars show mean ± SD and points show three independent-experiment means after technical triplicates were averaged (biological n = 3). Metformin was used at 2 mM; the concentration and exposure duration of the insulin-plus-palmitate control were not documented in the retained source record. One-way ANOVA followed by Tukey HSD was performed within each extract series relative to palmitate-only; a Bonferroni-adjusted family threshold of α = 0.0125 was applied across the four extract blocks: ns, p ≥ 0.05; **p < 0.01; ****p < 0.0001. No inferential comparison among extracts was performed. Any percentage changes reported in the text represent percentage differences relative to the concurrent palmitate-only mean and are not recovery or restoration percentages.

Within the retained palmitate-associated dataset, all four extract series showed greater apparent extracellular glucose utilisation than palmitate-only at one or more concentrations. The numerical means differed among the sequential extracts, but no inferential between-extract ranking was established. Because the endpoint was calculated from extracellular glucose disappearance, it does not establish membrane glucose transport or intracellular utilisation. The retained records did not document concentration-specific extract-only glucose-oxidase controls; optical or chemical assay interference therefore remains possible.

## 4 Discussion

This screening study identified differences among sequentially prepared *C. cassia* stem-bark fractions in preliminary phytochemical fingerprints, cytotoxicity and apparent extracellular glucose utilisation. Ethanol produced the broadest pattern of qualitative class reactions, whereas n-hexane produced the largest observed apparent-extracellular-glucose-utilisation mean under both basal and palmitate-associated conditions. Because extraction order increased with solvent polarity, polarity, order and the changing composition of the residual plant material are confounded; the findings therefore cannot establish that solvent polarity caused the biological differences. Nor do the terpenoid- and steroid-consistent reactions identify the active constituents. One hypothesis for future testing is that the n-hexane fraction contained one or more lipophilic constituents that contributed to the observed response. This remains speculative unless supported by quantitative chemical identification and activity-guided fractionation (Rudraswamy et al., 2026; Heinrich et al., 2022).

Chemical characterisation remains the principal unresolved limitation. TLC and colour-reaction tests cannot identify or quantify cinnamaldehyde, coumarin or other constituents, and positive coumarin class reactions in the ethyl acetate, ethanol and aqueous fractions do not establish the identity or concentration of coumarin. This is particularly important because coumarin exposure from cassia cinnamon has recognised safety relevance (Zhang et al., 2019; Abraham et al., 2010). No conclusion about chemical standardisation, human exposure or safety can therefore be drawn from the present data. Quantitative, validated HPLC or GC-MS measurement of cinnamaldehyde and coumarin in every fraction is required before composition-activity or safety interpretations can be advanced and to meet the preferred ConPhyMP characterisation standard (Heinrich et al., 2022).

The basal dataset showed descriptive concentration-related patterns for all four fractions. However, only the insulin wells were retained as observed absorbance values in the supplied basal workbook; the non-insulin well-level entries had been mathematically reconstructed from reported summary statistics. The reconstructed values are not raw experimental observations and were not used for confirmatory inference. The basal results require confirmation from original plate-reader exports or repetition of the experiment. The retained palmitate-associated dataset was analysed using independent-experiment means rather than treating technical wells as independent observations. Palmitate exposure was associated with lower apparent extracellular glucose utilisation than the normal control. The insulin-plus-palmitate condition was retained as an assay-response control, but its concentration and exposure duration were not documented, and quantitative mechanistic comparison is therefore limited. Insulin signalling, GLUT4, Akt phosphorylation, AMPK, lipid accumulation, mitochondrial function and inflammatory markers were not measured. The condition is accordingly described as palmitate-associated metabolic stress rather than insulin resistance.

During continued palmitate exposure, the largest reported mean differed across the four sequential extract series. The *post hoc* selection of these numerical maxima is vulnerable to winner’s-curse bias and is used only as an exploratory descriptor; it is not a formal cross-extract statistical comparison, evidence of an optimum concentration or evidence that solvent polarity caused the responses. The separate within-block analyses do not estimate an extract × concentration interaction, and biological n = 3 limits precision. The response decline above the largest observed mean for several extracts also argues against assuming a simple monotonic concentration-response relationship. The functional responses must be considered alongside cytotoxicity without describing a therapeutic or definitive safety window. The retained n-hexane 72-h MTT profile declined below 50% at 6.25 μg/mL, close to the highest functional concentration of 5.00 μg/mL, although the functional exposure was shorter. Viability, cell number and protein were not measured during the exact 180-min exposure. In addition, concentration-specific functional DMSO-control results and extract-only glucose-oxidase interference controls were not available in the records supplied for this revision. Absent cell integrity, vehicle-effect and analytical-interference controls, the observed extracellular glucose disappearance cannot be assigned specifically to membrane transport and should not be treated as evidence of a therapeutic effect.

The study’s strengths include botanical authentication and voucher deposition, sequential extraction across four solvents, preliminary TLC and phytochemical screening, inclusion of basal and palmitate-associated conditions, pharmacological controls and analysis based on biological rather than technical replication for the retained palmitate dataset. Its principal limitations are biological n = 3; confounding of solvent polarity with extraction order; absence of validated quantitative cinnamaldehyde and coumarin measurement; indirect estimation of extracellular glucose utilisation; reconstructed non-insulin basal well-level values; absence of concurrent viability, cell-number or protein normalisation; absence of documented concentration-specific functional DMSO-control results and extract-only glucose-oxidase interference controls; incomplete documentation of the insulin-plus-palmitate condition and extraction details; lack of *mycoplasma* testing; and absence of direct transport, GLUT4 or insulin-signalling measurements.

Future work should first quantify cinnamaldehyde and coumarin in every sequential fraction using validated HPLC or GC-MS methods and provide fit-for-purpose chemical fingerprints. It should also recover and archive the original basal plate-reader exports or repeat the basal experiment; repeat the functional assay with concentration-matched DMSO controls; include concentration-specific cell-free extract controls and glucose spike recovery or an orthogonal glucose assay; measure viability, cell number or protein during the exact functional exposure; increase biological replication; and retain complete nonlinear-regression settings, Hill slopes, goodness-of-fit statistics and curve-level confidence intervals if fitted potency parameters are reported. Direct transport assays and measurements of GLUT4 translocation and insulin-signalling pathways are required before mechanistic claims can be made.

In conclusion, sequentially prepared *C. cassia* stem-bark fractions showed different reported MTT metabolic-viability and apparent extracellular glucose-utilisation profiles. These screening observations may assist in prioritising rigorously controlled follow-up work. Because quantitative cinnamaldehyde and coumarin characterisation was not performed, the endpoint was extracellular glucose disappearance, solvent polarity and extraction order were confounded, basal well-level values were reconstructed, and concurrent viability and key analytical controls were unavailable, the present data do not establish chemical composition-activity relationships, glucose uptake, a specific transport mechanism, insulin sensitisation, extract superiority, therapeutic efficacy or *in vivo* safety.

## Data Availability

The original contributions presented in the study are included in the article/supplementary material, further inquiries can be directed to the corresponding author.
